# Understanding vision and the brain

**Published:** 2017-03-03

**Authors:** Anand Moodley

**Affiliations:** 1 Consultant neurologist Greys Hospital and University of KwaZulu-Natal Pietermaritzburg, South Africa.

**Diseases which affect the visual pathway or the nerves to the eye muscles are often serious. This article summarises the anatomy and function of the 2nd, 3rd, 4th and 6th cranial nerves and the signs and symptoms which are important in making a correct diagnosis.**

**Figure:**
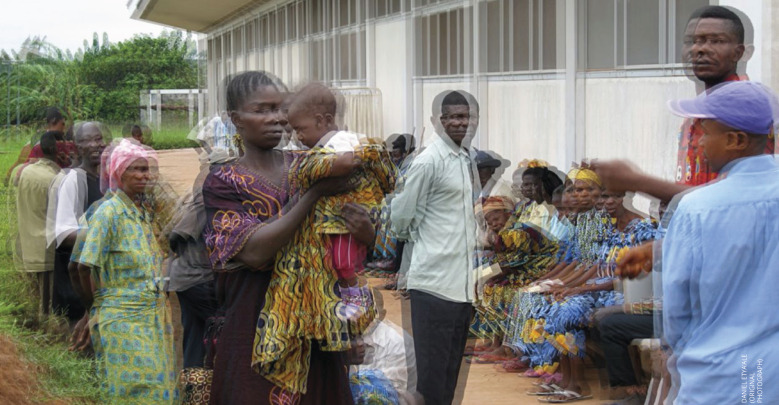
Double vision is a distressing symptom that warrants investigation. TANZANIA

Neuro-ophthalmology includes the specialties of neurology and ophthalmology. Skills of both specialties are needed to assess patients. A detailed history and clinical examination are needed in order to make a differential diagnosis and create a management plan.

There are two broad groups of neurological conditions that can affect someone's vision:

1. Those which affect the visual pathways in the brain and
2. Those which affect eye movements.

If the condition affects the visual pathways, there can be loss of visual acuity, loss of visual field, or difficulties in understanding the visual world, depending on where the lesion is in the visual pathway. If the condition affects the 3rd, 4th or 6th cranial nerves, the main symptom is double vision (diplopia) due to restricted eye movements.

**The visual pathways** comprise the optic nerve, optic chiasm, optic tract, optic radiation and the visual cortex in the occipital lobes.

Nerve impulses arising in the retina travel via the optic nerve to the optic chiasm. In the optic chiasm there is “crossing over” of some visual information, so that images which fall on the right visual field of each eye go to left visual cortex via the optic tract and the optic radiation. Visual information from the left visual field in each eye goes to the right visual cortex.

## Neurological conditions which affect visual function

Conditions which affect the visual pathways can lead to loss of visual acuity (ranging from blurred vision to complete visual loss), impaired colour vision, or visual field loss (which may be central, nasal or temporal) or affect the right or the left visual field. The type of visual field loss depends on which part of the visual pathway is affected.

### Visual Loss

Loss of central visual acuity, with impaired colour vision and contrast sensitivity, suggest an optic nerve condition.

The two main clinical presentations are:

- **Optic neuritis,** with a decrease in vision over a few hours or days and sometimes pain on eye movement from inflammation of the optic nerve and surrounding sheath. Visual acuity is decreased, there Is an afferent pupil defect (pupil on the affected side reacts poorly to light), and the optic disc may appear swollen (papillitis). It may be unilateral or bilateral. Causes include multiple sclerosis, some medications and toxins (e.g. cassava or methanol).
- **Optic atrophy,** in which the optic disc is pale. The visual acuity is reduced, colour vision is affected and there is an afferent pupil defect. There are many causes of optic atrophy (see page 69). Optic atrophy may develop slowly due to a progressive cause, or follow an episode of acute optic neuritis.

### Visual field defects

**Figure 1.**
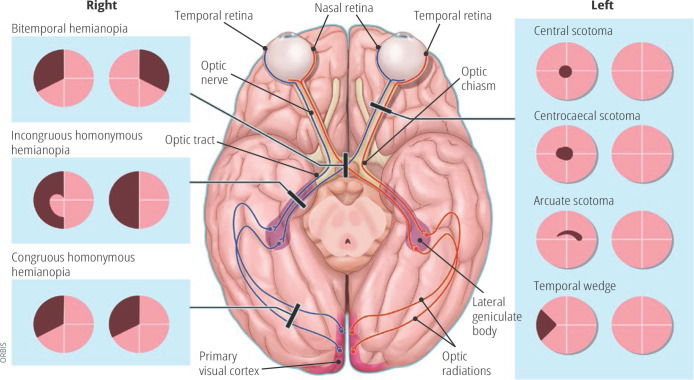
Types of visual field loss resulting from lesions in different parts of the visual pathway.

Lesions along the visual pathway, from the optic nerve to the occipital lobe, can present with localising visual field defects (**Figure 1**).

- **Optic nerve lesions** cause central, centrocaecal or altitudinal field defects. Central vision is primarily affected.
- **Optic chiasm lesions** caused by space-occupying lesions around the sella turcica (e.g. pituitary tumours) typically cause bitemporal hemianopia. Central vision is usually spared.
- **Optic tract, optic radiation and occipital lobe** lesions result in loss of vision in the opposite hemifield which patients experience as loss of vision on the right or left.

### Higher visual centres

Visual information goes from the visual cortex to the ‘visual association areas’ where what we see is interpreted. For example, visual information which goes from the occipital lobe to the temporal lobe enables faces and objects to be recognised, while visual information which goes from the occipital lobe to the parietal lobe allows objects to be localised in space.

## Neurological conditions which affect eye movement (See Figure 1 on page 64)

Diplopia (double vision) is the commonest symptom and results from misalignment of the visual axes in the eyes.

- Disorders causing diplopia may arise due to lesions affecting any part of the 3rd, 4th and 6th cranial nerves, or due to diseases affecting the extra-ocular muscles, e.g. myasthenia gravis or dysthyroid eye disease.
- Diplopia is experienced as either images lying side by side (horizontal diplopia) from involvement of the medial and lateral rectus muscles, or images lying one above the other (vertical diplopia) from involvement of the eye elevators.
- Separation of images is greatest in the direction of the action of the weak muscles and the false image created by the affected eye is usually the outermost image.

## Associated neurological symptoms

- Facial numbness (especially over the forehead), occurring in conjunction with diplopia from 3rd, 4th or 6th cranial nerve lesions, suggests disease at the cavernous sinus or orbit.
- Impaired vision on the same side of 3rd, 4th and 6th cranial nerve lesions suggests an orbital apex lesion.
- Weakness of the lower half of the face, arm and leg unilaterally (hemiparesis) occurring with 3rd or 6th nerve palsy (diplopia) suggest a lesion of the brainstem.
- Dysphagia (difficulty swallowing), dysarthria (slurred speech) with fluctuating double vision and eyelid drooping (ptosis) is suggestive of myasthenia gravis; a disorder of the neuromuscular junction.
- Patients with thyroid eye disease may have diplopia, proptosis, lid retraction (staring gaze) and an enlarged thyroid gland from Grave's disease.

